# Descriptive analysis of sickle cell patients living in France: The PHEDRE cross-sectional study

**DOI:** 10.1371/journal.pone.0248649

**Published:** 2021-03-18

**Authors:** Marie Gerardin, Morgane Rousselet, Marie-Laure Couec, Agathe Masseau, Marylène Guerlais, Nicolas Authier, Sylvie Deheul, Anne Roussin, Joelle Micallef, Samira Djezzar, French Addictovigilance Network (FAN), Fanny Feuillet, Pascale Jolliet, Caroline Victorri-Vigneau

**Affiliations:** 1 Service de Pharmacologie Clinique, Centre Hospitalier Universitaire de Nantes, Nantes, France; 2 Service d’Addictologie et de Psychiatrie, Centre Hospitalier Universitaire de Nantes, Nantes, France; 3 INSERM U1246 SPHERE “Methods in Patient-Centered Outcomes and Health Research”, Universités de Nantes et Tours, Nantes, France; 4 Service de Pédiatrie et d’Oncologie Pédiatrique, Centre Hospitalier Universitaire de Nantes, Nantes, France; 5 Service de Médecine Interne, Centre Hospitalier Universitaire de Nantes, Nantes, France; 6 Service de Pharmacologie Médicale, Centre Hospitalier Universitaire de Clermont-Ferrand, Clermont-Ferrand, France; 7 Service de Pharmacologie, Centre Hospitalier Universitaire de Lille, Lille, France; 8 Service de Pharmacologie Médicale et Clinique, Centre Hospitalier Universitaire de Toulouse, Toulouse, France; 9 Service de Pharmacologie Clinique, Hôpital de la Timone, Assistance Publique-Hôpitaux de Marseille, Marseille, France; 10 Centre d’Evaluation et d’Information sur la Pharmacodependence—Addictovigilance de Paris, Hôpital Fernand Widal, APHP Paris, Paris, France; 11 Plateforme de Biométrie, Centre Hospitalier Universitaire de Nantes, Nantes, France; University of Cape Town, SOUTH AFRICA

## Abstract

**Background:**

Sickle cell disease (SCD) induces chronic haemolytic anaemia and intermittent vaso-occlusion that results in tissue ischaemia causing acute, severe pain episodes that can lead to frequent hospitalizations. These consequences can have repercussions on family, social, school and/or professional life. Here, we present some of the results of the PHEDRE study (Pharmacodépendance Et DREpanocytose—drug dependence and sickle-cell disease), which is the largest study of patients with SCD in France. This paper intends to describe characteristics of the French SCD population. We also aimed to assess the impact of the disease on the lives of patients using objective and subjective variables.

**Methods:**

The PHEDRE study was a national multicentric observational study. Adults, adolescents and children with a confirmed SCD diagnosis were included in the study by their referring doctor. Then, they were interviewed by phone about their socioeconomic status, about the impact of the disease on their lives and about their analgesic and psychoactive drug use.

**Results:**

The study population consisted of 872 patients (28% were minors). Seventy-two percent of adults were active, and all minors were in school. Many patients presented criteria of severe SCD. Seventy-five percent were homozygous SS, 15% were double heterozygotes SC and 8% were heterozygotes Sβthal, 87% received specific treatment, 58% were hospitalized at least once for vaso-occlusive crisis in the past 12 months, and the number of analgesic drugs taken averaged 3.8. Seventy-five percent of patients reported academic or professional consequences related to their SCD, and 52% reported social consequences.

**Conclusions:**

The impact of SCD on patients’ lives can be significant, nevertheless their social integration seems to be maintained. We highlighted respect of recommendations regarding analgesic treatments and only a few patients used tobacco, alcohol or cannabis.

**Trial registration:**

Clinical Trials, NCT02580565; https://clinicaltrials.gov/ Registered 16 October 2015.

## Introduction

### A severe disease with serious consequences

Sickle cell disease (SCD) is an inherited red blood cell disorder. It is among the most common monogenetic diseases worldwild [1]. It is caused by homozygosity for a single amino acid change in the β-globin chain that results in structurally abnormal haemoglobin S (HbS), or by compound heterozygosity for HbS and another β-globin chain abnormality, typically haemoglobin C (HbC) or βthalassemia (Hbβthal) [1]. These mutations are structural variants of normal adult haemoglobin (HbA). Homozygous (SS individuals) who have inherited HbS alleles from both parents or compound heterozygotes individuals (SC for example) suffer from SCD, which often leads to acute and chronic complications [2,3]. Carriers or heterozygotes (AS individuals) can be asymptomatic but they may suffer from increased complications (like venous thromboembolism) compared to non-carriers [4]. The major characteristics of SCD are chronic haemolytic anaemia, vasculopathy and intermittent vaso-occlusion that results in tissue ischaemia causing acute, severe pain episodes that can lead to frequent hospitalizations. The intensity, repetition, anxiety and unpredictability of painful crisis lead to major functional and psychological impacts [5,6]. These consequences can have repercussions on family, social, school and/or professional life. Psychological complications have been identified in both children and adults with SCD [5,6].

### A prevalence that is difficult to estimate despite the evolution of the screening process

The lack of reliable data in most countries makes it difficult to estimate the actual number of affected people worldwide [7–12]. There are over 300,000 affected births each year, with a high frequency of patients with SCD across most of sub-Saharan Africa, Saudi Arabia, and India, as well as in descendants from the Caribbean, Central America, South America, and Mediterranean countries [13].

In France, SCD is considered a rare disease because it affects less than one person out of 2,000 [14]. There is no national registry for SCD in France and the prevalence in the general population, such as that of carrying the sickle cell trait, is unknown. In 2014, the total population of sickle cell patients (homozygous and compound heterozygotes) was estimated about 10,000 people [7]. In 2015, 466 children were born with SCD, i.e., a prevalence of one child affected per 1736 births. However, this figure makes it the most common genetic disease in France. This prevalence is much higher in the overseas departments (Guadeloupe, Martinique, French Guiana, Reunion and Mayotte) (1/499) and in the Paris region (1/765), where the populations at risk are concentrated [15].

Since 1999–2000, screening for SCD has been carried out in all new-borns in overseas departments. In metropolitan France, it is carried out in new-borns whose parents come from regions at risk [7,16].

Neonatal screening makes it possible to determine the child’s genotype (HbAS, HbSS, HbSβthal, SC or others) so that preventive treatment of anaemia and infectious complications can begin as soon as possible [15].

Newcomers or certain mildly symptomatic patients who have not had vaso-occlusive complications may be diagnosed late, particularly during pregnancy, during routine screening for at-risk populations, or in the event of an acute vaso-occlusive event or an ophthalmological or surgical complication [17].

### An early management of the disease

In France, patients with SCD are managed via a patient-oriented care network, especially Reference Centres or Special Centres for Children and Adults (RSCCA). The size of the active file varies greatly from one centre to another: from 2 patients to more than 3500 patients. At the first consultation of the new-born child, a specialist doctor in the management of SCD (in RSCCA) examines the new-born child and organizes with the parents the arrangements for the child’s medical and social care. The care recommendations from the French High Authority of Health (Haute Autorité de Santé - HAS) involve the organisation of a care network around the child (nurses and doctor in nursery, in school, at the child’s local hospital, paediatrician or general practitioner…). Surveillance visits, vaccinations, growth monitoring, preventive medicine advice are identical to those for the general paediatric population. The rhythm of medical visits during the first 2 years follows the vaccination schedule. Beyond that, a quarterly rhythm is recommended. A specific management for SCD is added to the general paediatric management after consultation between the attending physician and the physician specialized in the management of SCD. Once a year, a check-up is recommended, which can be performed in a day hospital to detect and treat specific complications of the disease at an early stage. The content of the check-up varies according to the age of the child and the clinical context. It is recommended that the factors that contribute to painful vaso-occlusive crisis (VOC) are explained to the parents and that they become educated in the initial management of a painful VOC. Therapeutic education should be offered to the child to familiarize with the management of his or her disease. It should be adapted to the child’s age and the clinical characteristics of SCD [16].

The management of adult SCD coordinated by a specialist doctor involves a large number of health professionals in the treatment and management of acute and chronic complications (internist, haematologist, general practitioner, emergency doctor, psychologist and psychiatrist, nurse, social workers) [18].

### Who are the SCD patients in France today?

For patients with SCD to be cared for and appropriately and effectively supported, it is crucial that health professionals have a comprehensive knowledge and understanding of how patients experience living with the condition, for example, through the use of patient-reported outcome (PRO) tools [19,20].

Here, we present some of the results of the PHEDRE study (Pharmacodépendance Et DREpanocytose—drug dependence and sickle-cell disease), the largest study of patients with SCD in France. This paper intends to precisely describe sociodemographic characteristics and context of the disease for patients included in the study: genotype, origin of the screening, status of siblings, we also aimed to assess the impact of the disease on the lives of patients using objective and subjective variables.

## Methods

### Study oversight

The PHEDRE study was a national observational, transversal, cross-sectional study conducted by the Nantes pharmacology addictovigilance department [21]. It was monitored by a pluri-disciplinary steering committee comprising pharmacologists, psychiatrists specialised in addiction, biostatisticians, pharmacists and specialists in SCD. This study was approved by the CCTIRS (Comité Consultatif sur le Traitement de l’Information en matière de Recherche dans le domaine de la Santé) on 11 March 2015, the CNIL (Commission Nationale de l’Informatique et des Libertés) on 11 June 2015 and the GNEDS (Groupe Nantais d’Ethique dans le Domaine de la Santé) on 3 March 2015. All participants received an information leaflet for their age range (as do the parents of minors) and provided written informed consent in accordance with the Declaration of Helsinki. For minors, parents also signed a consent form authorizing their children’s participation. The study is registered as Clinical Trials.gov ID: NCT02580565.

### Patients

To be eligible, patients had to present SCD diagnosis confirmed by genetic testing, regardless whether it is the homozygous or heterozygous form; be treated for SCD at a RSCCA participating in the study, regardless of the duration of the illness and give their written consent and written consent from one of the parents or legal guardians of minors (under 18).

State-protected adults (under guardianship) and subjects not having the general aptitudes to participate in the study assessment were excluded from the study.

### Study procedures

The complete description of study procedures is available in the publication of our study protocol [21]. Patient recruitment occurred where patients were treated, in RSCCA spread over metropolitan France and overseas departments, between September 2015 and December 2017. Their referring doctor included patients in the study and completed a subject’s medical data form. All patients were then interviewed by phone by a trained interviewer without practitioner opinions and without disruption to the patient-doctor relationship. Variables collected using the medical form and the phone interview are described in Table 1. An *ad hoc* standardized heteroquestionnaire for phone interview was validated by the steering committee. Four interviewers were trained to administer the telephone questionnaire. They were members of the research staff with scientific and clinical research background. Before starting phone interviews they had a specific formation on SCD and pharmacodependence with members of the steering committee. First interviews were supervised by the principal investigator until interviewers were able to conduct the phone interview alone.

**Table 1:** Variables collected in the PHEDRE study.

Variables	Collection of data	Subjects concerned
**Clinical data**		
• Genotype • Screening situation • Age at diagnosis • Age at beginning of specific care for SCD • Presence of SCD in siblings • Specific treatment for SCD • Number of hospitalizations for SCD in the last 12 months • Including number of hospitalizations for VOC in the last 12 months • Participation in therapeutic education programme • Other current health problems (somatic or psychic)	Medical form	All patients
**Socio-demographic data**		
• Age • Sex	Medical form	All patients
• Activity • Lifestyle	Phone interview	All patients with responses choice adapted to age
• Parent’s work	Phone interview	Minor patients
**Impact of SCD**	Phone interview	• All patients with questions adapted to age • And parents for minor patients
**Analgesics consumption in the last 12 months**	Phone interview	All patients
**Tobacco, alcohol and psychoactive drug consumption**	Phone interview	Patients aged 12 years and over
**Psychoactive treatment used (INN)**	Phone interview	Patients aged 12 years and over

INN: International Non Proprietary Name.

All the medical data were collected by the referring doctor for SCD using the medical form. Genotype included homozygous SS or compound heterozygotes SC, Sβthal or others. Specific treatments for SCD included hydroxyurea, transfusion or transfusion exchanges, bone marrow transplant, bloodletting or other treatments.

Sociodemographic data were collected in part with the medical form (sex and age) and with phone interview (activity, lifestyle and parent’s work). Regarding employment activity, the possible responses for adult patients were “stable employment”, “precarious employment”, “student”, “disability”, or “no activity”. In France, disability is a status recognising a partial or total reduction in one’s capacity to work, as a result of injury or illness, and which may give entitlement to special benefits. Precarious employment includes employment contracts that are not open-ended and whose duration is therefore limited or undefined (temporary contracts, fixed-term contracts and assisted contracts) and certain jobs that do not pay enough to live decently (non chosen part-time jobs). For children, school activity included educational level. Regarding lifestyle, the possible responses for adults included “living with parents”, “living with at least one family member” (other than parents), “living alone” or “living in couple” (with or without children for each modality). For children, the possible responses were “living with both parents”, “living with one parent”, “living with another family member” (with or without siblings for each modality).

Perception of the impact of the disease (social, academic or professional consequences of the SCD) was evaluated as data reported by patients. Adult patients were asked two standardised questions: 1) about the consequences of their illness on their social life (in social interactions, leisure activities…) and 2) about the consequences of their illness on their professional (or academic for students) life (absenteeism, loss of employment, inability to work in certain occupations…). Children were asked two standardised questions: 1) about the consequences of their illness on their social life (in social interactions, leisure activities…) and 2) about the consequences of their illness on their school life (absenteeism, repetition, inability to participate in certain school activities…). The parent was also asked about the impact on the child’s life (consequences of illness on their child’s social life and consequences of illness on their child’s school life).

Patients were also addressed on analgesics consumption in the past 12 months. Data included the INN of analgesics and the context of consumption (at home or at hospital).

The other data collected were (only for patients aged 12 years and over), tobacco, alcohol and psychoactive drug consumption, and treatments used (prescribed or not prescribed).

### Outcomes

Our first objective was to describe the SCD population. We described the sociodemographic characteristics and the characteristics of the disease for patients included in the study (genotype, origin of the screening, status of siblings, other health problems).

Our other objective was to assess the impact of the disease on patients’ lives, using *(i)* objective outcomes, *i*.*e*., number of hospitalizations and type and number of treatments, and *(ii)* data reported by patients *i*.*e*., perception of the social and academic consequences of the disease reported by adult patients and either by minor patients and their parents.

### Statistical analysis

All evaluation parameters were subjected to a descriptive analysis. The quantitative variable evaluation parameters were described using position parameters (mean or median) and dispersion (standard deviation (SD), interquartile range (IQ)). The qualitative variable evaluation parameters are shown in the form of number and frequency tables for each modality.

## Results

All RSCCA in France (metropolis and overseas) were asked to participate in the study. With the exception of one centre, all of the main centres accepted to participate and included patients. Finally, 73 RSCCA participated, 37 centres for adults and 36 centres for children, in the whole French territory. 993 patients were initially included. A total of 121 patients were excluded because they were unreachable, did not meet the inclusion criteria or refused to answer the questionnaire. The study population consisted of 872 patients.

### Descriptive analysis

#### Socio-demographic data

**Fig 1:**
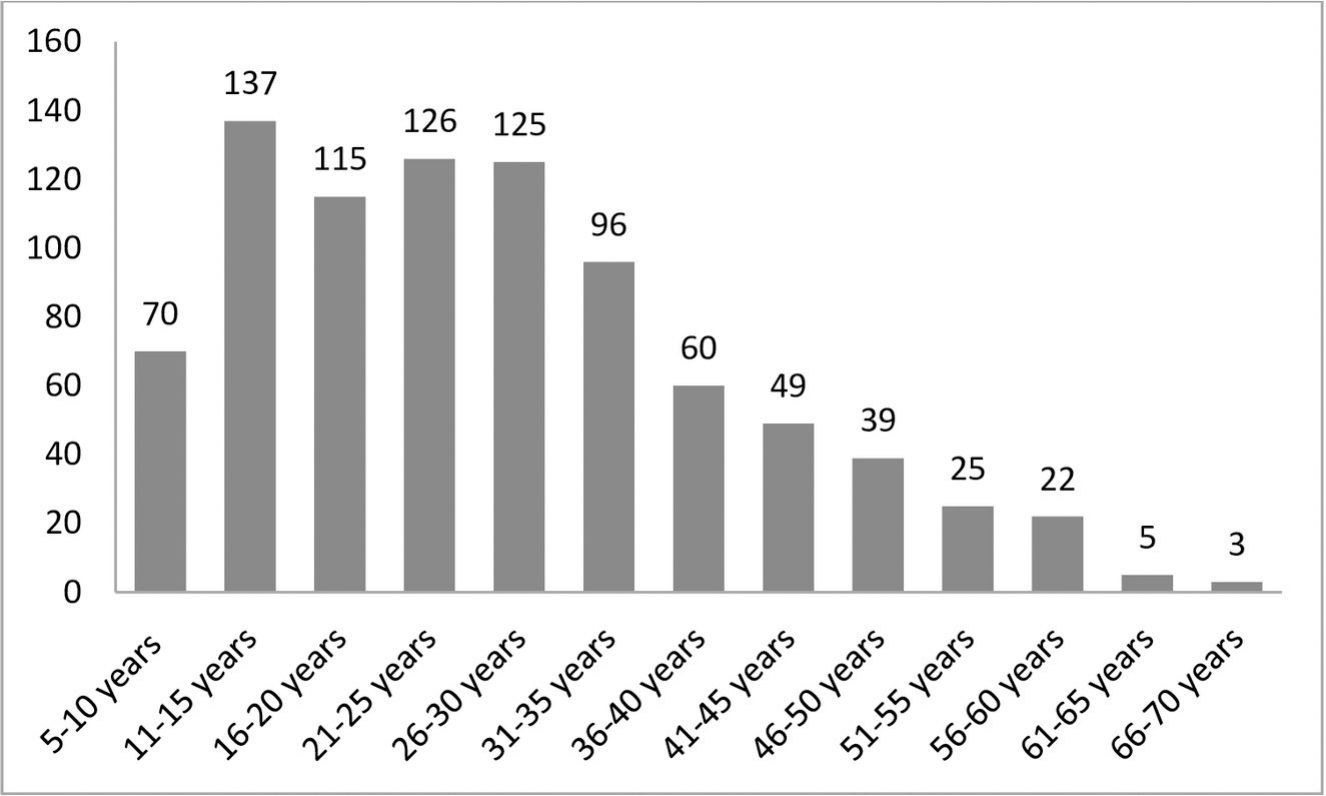
Age distribution.

A total of 503 patients were female (57.7%). The average age was 26.8 years (median: 25 years; min: 5 years—max: 69 years; SD: 13.19; IQ: [16.00;35.00]). A total of 625 were over eighteen (71.7%), and 247 were minors (28.3%). Fig 1 shows the distribution of subjects by age groups, presented in 5-year increments.

Among the adult subjects, 37 patients (5.9%) were disabled and 141 (22.6%) had no activity (neither professional nor student activity). 446 patients (71.5%) were active: 36.5% had stable employment, 26.0% were students and 9.0% had precarious employment.

A total of 163 adult patients (26.1%) were living with their parents or at least one family member, 195 were living alone (31.2%) and 204 were living in a couple (32.7%). A total of 209 patients (33.4%) had dependent children.

Among the 247 children and adolescents, 155 (62.8%) were living with both parents, 87 (35.2%) with one of the parents, and 5 (2.0%) with another person. At least one of the two parents was working in 85.0% of cases. All minors were in school. A total of 168 (69.1%) had an educational level in accordance with their age (N = 243), 62 (25.5%) were behind in school and 13 (5.4%) had an academic advance.

#### Clinical data

Clinical data concerning SCD and associated diseases are presented in Table 2.

**Table 2:** Clinical data.

		ADULTS N = 625	MINORS N = 247	Total N = 872
SCD genotype	N missing data	0	1	1
	N	625	246	871
	Homozygous SS	465 (74.40%)	192 (78.05%)	657 (75.43%)
	Heterozygotes SC	109 (17.44%)	25 (10.16%)	134 (15.38%)
	Heterozygotes S-βthal	45 (7.20%)	26 (10.57%)	71 (8.14%)
	Other heterozygotes	6 (0.96%)	3 (1.22%)	9 (1.03%)
Screening situation	N	625	247	872
	Neonatal screening	216 (34.56%)	187 (75.71%)	403 (46.22%)
	VOC	248 (39.68%)	31 (12.55%)	279 (32.00%)
	Other situation	161 (25.76%)	29 (11.74%)	190 (21.79%)
Existence of other SCD in	N missing data	20	6	26
Siblings	N	605	241	846
	No siblings	100 (16.53%)	63 (26.14%)	163 (19.27%)
	At least one sick sister/brother	243 (40.17%)	77 (31.95%)	320 (37.83%)
	Only patient of the siblings	262 (43.31%)	101 (41.91%)	363 (42.91%)
Participation in a therapeutic	N missing data	5	4	9
education programme (patient,	N	620	243	863
entourage)	No	531 (85.65%)	168 (69.14%)	699 (81.00%)
	Yes	89 (14.35%)	75 (30.86%)	164 (19.00%)
Other current health problems	N	625	247	872
(somatic or psychic)	No	423 (67.68%)	205 (83.00%)	628 (72.02%)
	Yes	202 (32.32%)	42 (17.00%)	244 (27.98%)

The mean age at the time of diagnosis was 3.9 years (median: 0 years; min: 0 years—max: 54 years; SD: 7.33; IQ: [0.00; 4.00]). The average age at which care began was 12.0 years (median: 8 years; min: 0 years—max: 61 years; SD: 12.77; IQ: [0.00; 21.00]).

#### Psychoactive substance use

751 patients aged 12 years and over were asked about their use of psychoactive drugs (other than analgesics) or substances.

Concerning medication (N = 745; 623 adults, 122 minors), a total of 111 patients (14.9%) reported the use of psychoactive drugs: 108 adults (17.3%) and 3 minors (2.5%).

Among adults, 68 patients cited hydroxyzine (10.9%), 24 cited benzodiazepines and related drugs (3.9%) and 20 cited antidepressants (3.2%). Two adult patients reported the use of phenobarbital.

Only 3 patients aged 12 to 17 years reported the use of psychoactive medications. All three subjects cited hydroxyzine.

With regard to psychoactive substance use (N = 743), 274 patients cited alcohol (36.9%), 99 cited tobacco (13.3%) and 43 cited cannabis (5.8%). No patients consumed alcohol on a daily basis. Smoking was a daily occurrence in 66 patients (68.0% of the smokers, N = 97) and cannabis in 19 patients (45.2% of the cannabis users, N = 42).

### Impact of the disease on the patient’s life

#### Objective variables

Table 3 presents the impact of SCD on patients in terms of care needs.

**Table 3:** Impact of SCD on patients—care needs.

		ADULTS N = 625	MINORS N = 247	Total N = 872
Number of hospitalizations related	N	625	247	872
to SCD in the previous 12 months	No hospitalization	213 (34.08%)	81 (32.79%)	294 (33.72%)
	1 or 2 hospitalization(s)	228 (36.48%)	102 (41.30%)	330 (37.84%)
	3 or more hospitalizations	184 (29.44%)	64 (25.91%)	248 (28.44%)
Including number of hospitalizations	N	625	247	872
related to VOC	No hospitalization	266 (42.56%)	102 (41.30%)	368 (42.20%)
	1 or 2 hospitalization(s)	216 (34.56%)	98 (39.68%)	314 (36.01%)
	3 or more hospitalizations	143 (22.88%)	47 (19.03%)	190 (21.79%)
Specific treatment for SCD	N missing data	1	0	1
	N	624	247	871
	No	74 (11.86%)	44 (17.81%)	118 (13.55%)
	Yes	550 (88.14%)	203 (82.19%)	753 (86.45%)

For all patients, the average number of hospitalizations related to SCD in the last 12 months was 1.97 (SD: 2.57), including 1.52 hospitalizations for VOC (SD: 2.15; min: 0 –max: 20).

Among the 753 patients receiving specific treatment for SCD, 590 received transfusion or transfusion exchange (78.4%), 481 were treated with hydroxyurea (63.9%), 141 received bloodletting (18.7%), 10 received a bone marrow transplant (1.3%) and 63 received other treatments (8.4%).

All patients had taken at least one analgesic drug in the last 12 months The number of analgesic drugs taken in the last 12 months at home and/or in the hospital averaged 3.8 (SD: 1.72).

At home (N = 870), 778 patients (89.4%) used grade 1 analgesics, 669 patients (76.9%) used grade 2 analgesics, and 73 patients (8.39%) used grade 3 analgesics. 714 patients (82.1%) used only grade 1 analgesics or grade 1 and grade 2 analgesics. 92 patients (10.6%) didn’t use any grade 1 analgesics.

**Fig 2:**
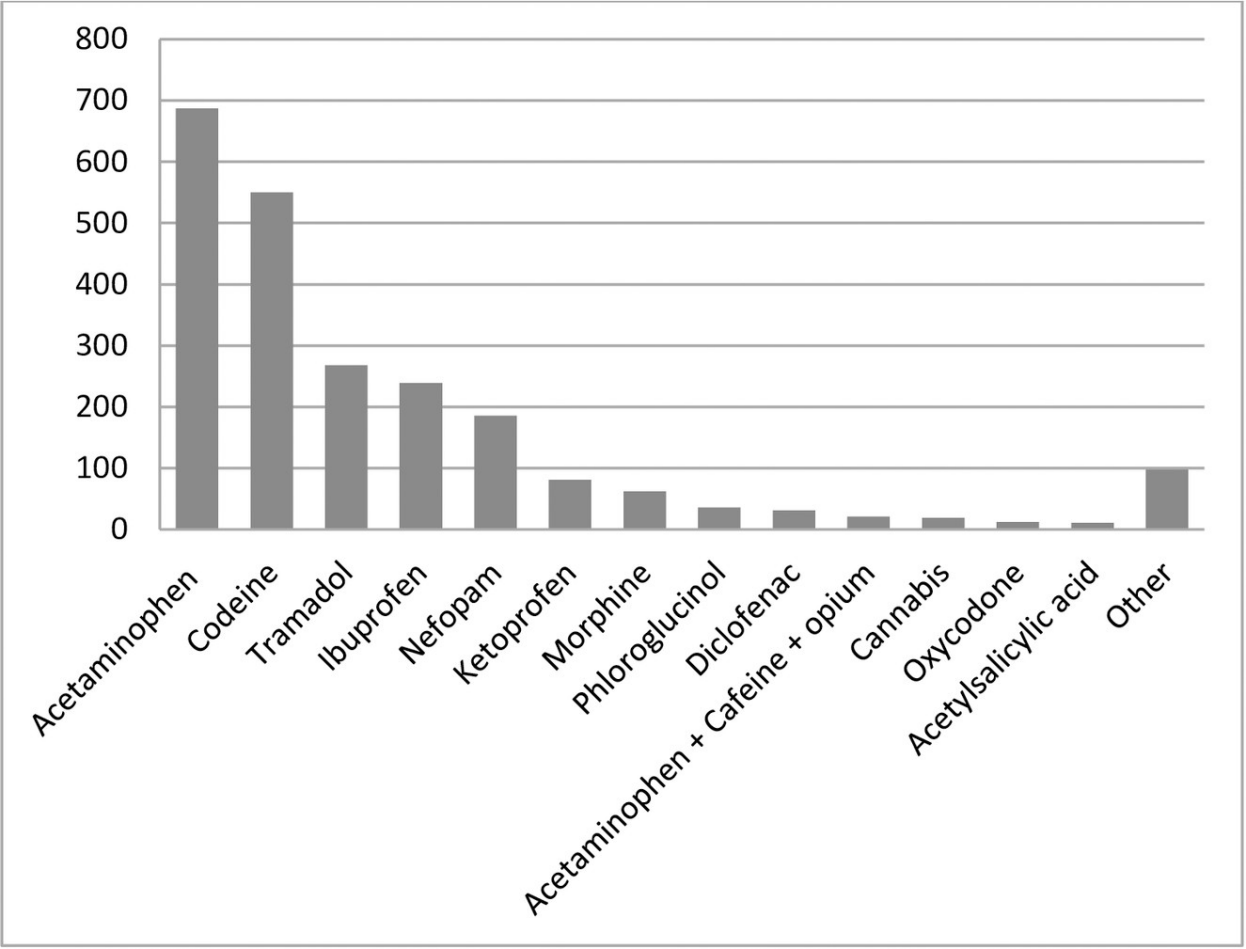
Analgesic drugs used at home.

The drugs most commonly used by patients at home were acetaminophen, codeine in combination, NSAIDs and tramadol. Fig 2 lists the substances used for analgesic purposes taken by patients at home in the last 12 months. The percentage is calculated based on the total number of patients (n = 872).

#### Subjective variables reported by patients

A total of 643 patients (74.5%) reported academic or professional consequences related to their SCD (N = 863), and 449 (52.1%) reported social consequences (N = 872).

Regarding academic consequences, the children (N = 240) answered “yes” in 53.3% of cases (difficulties in taking courses, absenteeism, repeating a year) and their parents (N = 129) answered "yes" in 61.2% of cases. Regarding social consequences, the children (N = 241) answered “yes” in 48.1% of cases (inability to participate in certain leisure and/or social activities or to go out under certain weather conditions) and their parents (N = 129) answered "yes" in 51.9% of cases. The differences were not significant.

## Discussion

### Strengths and limitations of the study

We included 993 patients in the study, which represents nearly 10% of the SCD population in France (about 10,000). Investigating physicians reported difficulties to include more patients, such as insufficient time and insufficient human resources. Others have highlighted the large number of clinical studies involving patients with SCD and the impossibility of including all patients completing inclusion criteria in our study.

Moreover, we assume that there is certainly a bias in the selection of patients included. Physicians probably offered the study more readily to patients whom they thought might be willing to answer a telephone questionnaire, and perhaps to patients who were problematic in their management. Due to the study design, and the need for the subjects included to be able to understand and answer questions asked by telephone, no children under the age of 5 were included neither people who were not fluent in French language (particularly in overseas departments).

However, the PHEDRE database is an important data source concerning SCD patients. Patients were interviewed directly to gather their feelings about how they live with their condition and about their use of medication and psychoactive substances. The data collected in our study allowed a description of the French SCD population that has never been published before.

### Description of socio-demographic characteristics

The socio-economic data of the patients included in the study corresponded to the characteristics of the French national population [22–24], with the exception of the inactivity rate of the adult subjects. Indeed, the inactivity rate seems higher than in the general population (22.6% versus 9 to 10%, respectively, in France) [22] but lower than in other studied SCD populations (40 to 60% in the USA) [25,26]. That said, we cannot truly compare the socio-professional data of French SCD patients from the study with those in the literature. Indeed, SCD patient populations around the world live in very different conditions depending on the country, even between developed countries.

The cause of unemployment in SCD is poorly understood, but it is likely that SCD plays a role in patient difficulties in finding or keeping a job. On the one hand, painful VOC can lead to significant absenteeism, and on the other hand, certain working conditions that may represent favourable factors for VOC are incompatible with patients’ pathology. Williams *et al* [26] found that employed patients had decreased encounters of health care services compared with unemployed patients. Other studies suggested that employment status may not be related to health but to behavioural and social factors [27].

All the school-age children were enrolled in school. The educational delay rate (25.5%) was lower than in the French general population (33.9% between the ages of 7 and 18 in 2005) according to data from the National Institute of Statistics and Economic Studies (Institut National de la Statistique et des Etudes Economiques; INSEE) [24]. However, these data should be interpreted with caution because, in our study, the question was asked of the children at time T, and the data were not available at the end of schooling, so we could not calculate the actual delay rate. A study has shown that, in some countries, SCD children and adolescents had a lower level of education than the national average, particularly due to the social difficulties faced by the black ethnic groups to which these patients belong [28]. However, the study data did not include European patients.

In other studies, sickle pain was reported to negatively impact school attendance and functioning, sleep patterns, daily and social activities, health service use and health-related quality of life [19,29–33]. These studies highlighted that SCD could affect abilities of children and adolescents to concentrate because of fatigue and led to frequent school absenteeism due to symptoms, hospitalization or medical appointments. Children and adolescents included in the PHEDRE study reported such difficulties but it seems that they do not have an impact on the smooth running of their schooling.

### Screening situation

The mean age at the time of diagnosis was 3.9 years. This can be explained by the presence in the study population of subjects born prior to the introduction of current neonatal screening modalities.

Three-fourths of the patients included in the PHEDRE study were homozygous genotype SS. There are no data available in France on the prevalence of one genotype compared to the other, but the prevalence of heterozygote patients is much higher than that of homozygous patients [3,7,34]. However, since homozygous patients are often more symptomatic [7,35], they are logically more likely to have been included in the study. Another result seemed to confirm that the study population was made up of patients with rather severe SCD: 86.5% of the patients received specific treatment for SCD. The two most frequently cited treatments were transfusion programmes and hydroxyurea. According to French guidelines, these two treatments are the only disease-modifying treatments for SCD currently validated but they carry risks of complications for patients. Approximately 30% to 40% of homozygous SCD patients (or patients with Sβthalassemia) have a hydroxyurea indication if they have at least one of the following two criteria: three hospitalizations in one year for VOC or a severe or recurrent acute chest syndrome [5]. Its effectiveness can be spectacular in some patients. This treatment reduces the number of bone VOC, acute thoracic syndromes, transfusions and hospitalizations but difficulties in compliance are a frequent cause of clinical inefficiency [36,37].

Nearly 1 in 3 patients reported suffering from at least one other health problem (somatic or psychological). We have not received the details of the health problems reported by the patients, but given the mentioned psychoactive drugs taken, we can assume a small proportion of patients with psychological disorders such as depression and anxiety.

### Impact of the disease on the patient’s life

A total of 57.8% of patients had been hospitalized at least once for VOC in the previous 12 months. The number of hospitalizations per patient was variable but can be very high: at least 3 hospitalizations for more than one in 5 patients and up to 20 hospitalizations in the last 12 months. Risk factors for hospital admissions and readmissions (within a 30-day period) have been described. Clinical manifestations of VOC do not solely contribute to rates and lengths of hospitalisation [26,38,39]. Risk factors included age (adolescents more than adults and adults more than children), living in low socio-economic areas, unemployment, and comorbidities such as asthma or depression. Moreover, low-hospital-using patients would be rational actors in the control of their disease.

Data reported by patients are important data to collect in patients with SCD. Indeed, patients’ poor perception of quality of life can lead to depressive symptoms, which are themselves risk factors, particularly in children, for chronic pain [40,41].

In our study, half of the subjects reported social consequences. SCD patients may be confronted with a lack of understanding among those around them, particularly regarding painful crisis (frequency, intensity and impossibility of carrying out certain activities), due to a lack of knowledge of the disease [42]. In their study conducted in the UK, Chakravorty *et al* showed how patients perceived the impact of SCD on daily life: more than half cited everyday activities and professional or academic consequences, one third cited people’s attitudes, and one fifth cited socialization problems [42].

Freitas *et al* showed that certain parameters were associated with lower scores on quality of life in adults with SCD, notably, frequent painful crises, severe forms of SCD, frequent hospitalizations, comorbidities or complications and mood disorders [43].

Three out of four patients included in the PHEDRE study reported academic or professional consequences related to their SCD and half reported social consequences. These difficulties could explain to a certain extent the high proportion of adults with no professional activity.

For minor subjects, our results showed no repercussions of SCD in terms of academic delay, but the consequences for children’s schooling and quality of life are real, as stated above [19,29–33]. A Dutch study compared the health-related quality of life (HRQoL) of children with SCD to that of healthy siblings [44]. The results showed that the HRQoL of children with SCD appeared similar to the HRQoL of healthy siblings, with the exception of lower scores on the physical domain for children with SCD. Surprisingly, children with SCD did not report any social problems. However, the HRQoL of both children with SCD and healthy siblings was considerably lower than that of the Dutch norm population in several domains. This implied that reduced HRQoL in children with SCD is primarily related to the low socio-economic status of this patient population.

In our study, the answers concerning the perception of the impact of the disease on the child’s life were consistent between the children and parents. The parents tended to report a greater impact of the disease on their children’s lives than their children themselves, but the difference was not significant. Other studies have shown that parents of chronically diseased children generally tend to report worse HRQoL for their children than children themselves [45,46].

Goldstein-Leever *et al* have shown a link between parents’ perception of pain and children’s perception of pain in SCD. Parents’ catastrophizing about their child’s pain contributed to child’s depressive symptoms, symptoms that have been identified as risk factors for chronic SCD pain [41]. Parental health and parenting beliefs and behaviours are known to have a significant impact on child pain management, and parental emotional distress and depression represent an independent risk factor for adverse child outcomes. This highlights the value of depressive symptom screening within the paediatric SCD population. It is also important to assess the quality of life of the parents who take care of the children, as SCD also impacts close family members of patients [41,47].

### Medication and psychoactive substances use

Only 15% of patients reported the use of psychoactive drugs other than analgesics. Hydroxyzine was cited 71 times. This drug is usually used as adjuvant therapy in painful crisis for an anxiolytic effect [5]. Only 24 patients cited treatment by benzodiazepines (3.9% of adults). This percentage seems low in view of the consumption of benzodiazepines in the general French population (13% in 2015) [48], but according to French guidelines, benzodiazepines are to be avoided in combination with opioïds, as benzodiazepines can promote respiratory depression [5]. We cannot deduce from these results the percentage of patients suffering from psychological disorders. In other studies, the proportion of SCD patients reporting psychological disturbances was very variable: 18 to 62% for depressive symptoms and 7 to 67% for anxiety symptoms [26,49–52].

Two people reported taking phenobarbital treatment. In both cases, it had been used to prevent yellow discoloration of the eyes due to jaundice. This practice is outside the marketing authorisation but seems to be known to doctors in reference to SCD because of its enzymatic inducer properties, which accelerate the degradation of bilirubin.

The use of alcohol, tobacco and cannabis in the subjects included in the study was very low. Only 43 patients (5.8%) cited marijuana. The absence of tobacco and alcohol consumption is part of the basic hygieno-dietary rules in patients with SCD [5]. Cannabis is used by some chronic pain patients to relieve pain, including patients with SCD [53]. However, it seems that French patients included in the PHEDRE study make little use of this substance.

According to the French guidelines, analgesics are systematically prescribed at each consultation, allowing the patient to have them at his disposal at all times. Grade I or grade II analgesics can be used depending on the intensity of the pain. Grade II analgesics should only be taken if pain persists despite the use of grade I analgesics; indeed, if codeine derivatives are used repeatedly to prevent pain, this misuse leads to a risk of addiction and side effects. Grade III (morphine) analgesics should not be prescribed in outpatient settings [5].

At home, according to our results, most patients used grade I analgesics (most often acetaminophen), often combined with grade II analgesics (most often codeine). Few patients used grade III analgesics (8.4%), most often morphine. The treatments for the majority of patients included in the study (82.1%) respected recommendations.

Ten percent of patients admitted to not using any grade I analgesics. SCD patients are well aware of the warning signs of a painful attack, and some patients explained that they would directly take a grade II analgesic when anticipating an intense painful attack, thinking that grade I analgesics would not be effective.

In conclusion, the population of SCD patients included in the PHEDRE study presents similar characteristics with SCD populations described in other countries, but their social integration seems better according to objective criteria. SCD, however, seems to be severe for most patients interviewed, and the patients reported a great impact of the disease on their daily life is real. Objective data confirmed that they are often hospitalized, use many painkillers as well as psychotropic drugs, have comorbidities and may encounter professional and/or social difficulties. Further studies are needed to assess the role of social status in the difficulties reported by patients in France.
